# P-1046. Burkholderia Bloodstream Infections In Indian Hospitals: A Multicenter Analysis Of Clinical Burden And Outcomes

**DOI:** 10.1093/ofid/ofaf695.1241

**Published:** 2026-01-11

**Authors:** Parul Singh, Nizamuddin Ahmed Mohammed, Arpan Kumar, Mamta Purswani, Ransa Parveen, Ashish Srivastava, Kapil Soni Dev, Kamraan farooque, Purva Mathur

**Affiliations:** AIIMS,New Delhi, New Delhi, Delhi, India; AIIMS New Delhi, Delhi, Delhi, India; AIIMS New Delhi, Delhi, Delhi, India; AIIMS New Delhi, Delhi, Delhi, India; AIIMS New Delhi, Delhi, Delhi, India; AIIMS New Delhi, Delhi, Delhi, India; AIIMS,New Delhi, New Delhi, Delhi, India; AIIMS,New Delhi, New Delhi, Delhi, India; AIIIMS ,New Delhi, New Delhi, Delhi, India

## Abstract

**Background:**

Burkholderia species are emerging as significant pathogens in nosocomial bloodstream infections, particularly central line-associated bloodstream infections (CLABSIs). These infections present a clinical challenge due to their association with invasive devices and their prevalence in critical care settings. Understanding the epidemiological trends and outcomes is vital for strengthening infection prevention strategies and resource planning in Indian healthcare facilities.

**Figure:**
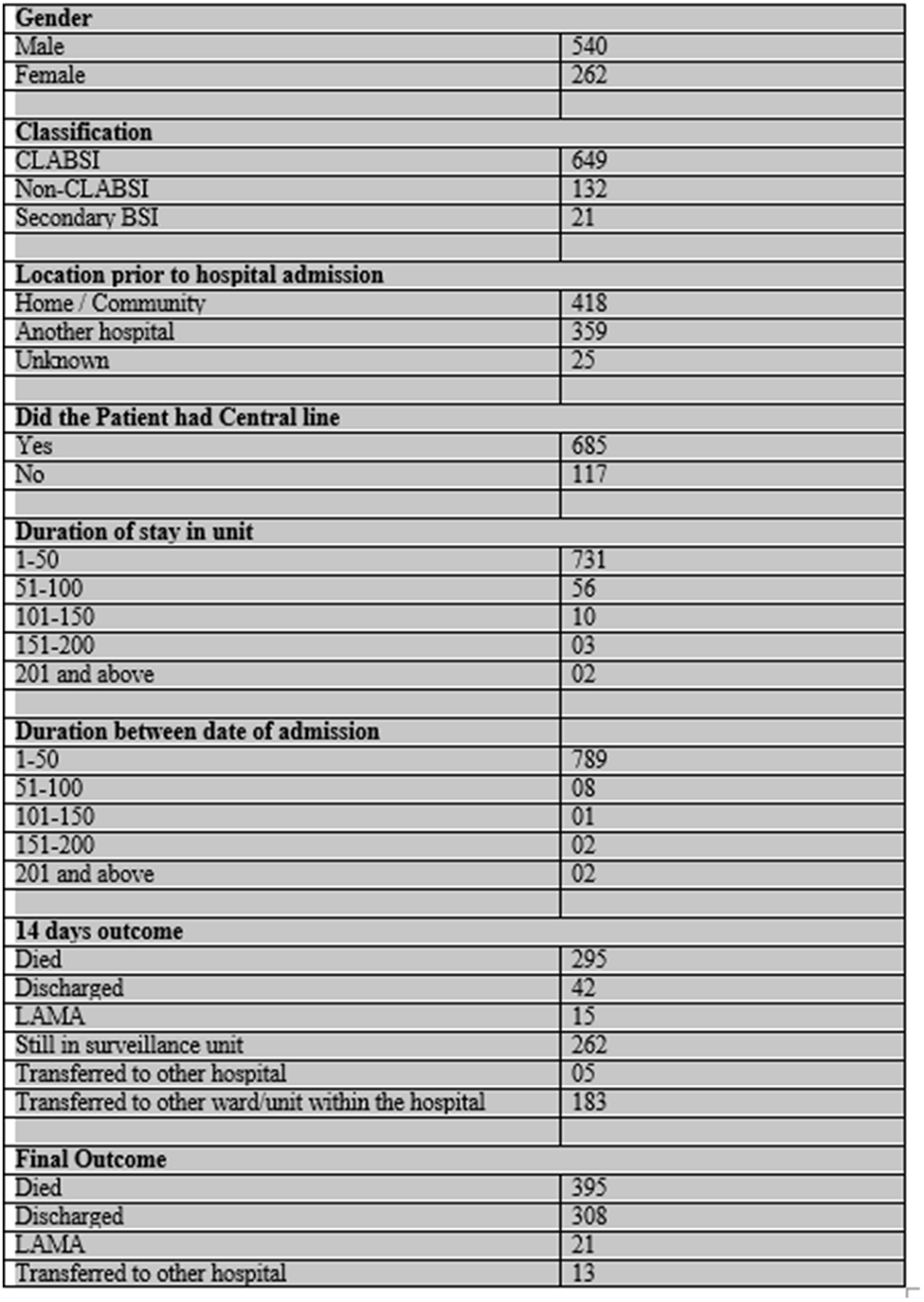
Demographic, Clinical, and Outcome Profile of Patients with Burkholderia Bloodstream Infections (2017–2024)

**Methods:**

This retrospective multicenter study included 802 confirmed cases of Burkholderia bloodstream infections reported between 2017 and 2024 across hospitals under the Indian Council of Medical Research (ICMR) and National Centre for Disease Control (NCDC) networks. Data on demographics, infection type, hospital setting (public vs. private), admission source, clinical outcomes, and unit distribution were analyzed. The study encompassed multispecialty, trauma, and oncology hospitals from various geographic regions of India.

**Results:**

Among the 802 patients, 67.3% were male, and 85.5% had a central line. CLABSI was the most common infection type (81%). Patients were primarily admitted from the home/community (418 cases), followed by referrals from other hospitals (359). The majority of infections (82%) were reported from public hospitals, with the rest from private institutions. Burkholderia cepacia was the predominant organism isolated (728 cases), with smaller numbers of B. pseudomallei, B. cenocepacia, and others. During the 14-day surveillance period, 295 patients (36.8%) died, while the final outcome data showed a mortality of 49.2% (395 patients). A total of 308 patients were discharged, 21 left against medical advice, and 13 were transferred to other hospitals. These findings reflect a high disease burden, especially in patients with central lines and those admitted to public sector hospitals.

**Conclusion:**

Burkholderia bloodstream infections, particularly CLABSIs, are associated with significant morbidity and mortality in Indian hospital settings, predominantly affecting patients in public sector multispecialty units. These findings suggest centralized monitoring to reduce the burden of these healthcare-associated infections.

**Disclosures:**

All Authors: No reported disclosures

